# Asylum Journal

**Published:** 1842-12

**Authors:** 


*Asylum Journal.*—A quarto sheet is hereafter to be published weekly at the Insane Asylum in Brattleboro', Vt., *by the inmates of the institution.* This is indeed an extraordinary age, when lunatics write for the amusement and instruction of the rest of mankind! Who can say, however, but that a large part of those who are outside the walls of insane hospitals are quite as crazy as those within? The contents of the first No. are creditable to the literary taste and morals of those who prepared it for the press. All the profits are to be applied to the support of the indigent residents of the asylum, of whatever name or denomination. The price is only one dollar a year, and those who profess to be the friends of unfortunate humanity cannot better appropriate that pittance than by forwarding it to Brattleboro' for the sustenance of this novel enterprise.—*Boston Med. and Surg. Jour.*

